# Early myocardial damage and microvascular dysfunction in asymptomatic patients with systemic sclerosis: A cardiovascular magnetic resonance study with cold pressor test

**DOI:** 10.1371/journal.pone.0244282

**Published:** 2020-12-22

**Authors:** Nicola Galea, Edoardo Rosato, Antonietta Gigante, Cristian Borrazzo, Andrea Fiorelli, Giovanni Barchetti, Amelia Chiara Trombetta, Maria Anna Digiulio, Marco Francone, Carlo Catalano, Iacopo Carbone

**Affiliations:** 1 Department of Experimental Medicine, Sapienza University of Rome, Rome, Italy; 2 Department of Radiological, Oncological and Pathological Sciences, Sapienza University of Rome, Rome, Italy; 3 Department of Clinical Medicine, Clinical Immunology Unit- Scleroderma Center, Sapienza University of Rome, Rome, Italy; 4 Statistical Unit, Department of Public Health and Infectious Disease, Sapienza University of Rome, Rome, Italy; 5 Radiotherapy Unit, Ospedale San Pietro Fatebenefratelli, Rome, Italy; Scuola Superiore Sant'Anna, ITALY

## Abstract

**Purpose:**

Cardiac involvement in Systemic Sclerosis (SSc) is increasingly recognized as a mayor cause of morbidity and mortality. The aim of present study is to investigate the early stages of cardiac involvement in SSc by Cardiovascular magnetic resonance (CMR), combining the non-invasive detection of myocardial inflammation and fibrosis using T2 and T1 mapping techniques and the assessment of microcirculatory impairment through perfusion response to cold pressor test (CPT).

**Methods:**

40 SSc patients (30 females, mean age: 42.1 years) without cardiac symptoms and 10 controls underwent CMR at 1.5 T unit. CMR protocol included: native and contrast-enhanced T1 mapping, T2 mapping, T2-weighted, cineMR and late gadolinium enhancement (LGE) imaging. Microvascular function was evaluated by comparing myocardial blood flow (MBF) on perfusion imaging acquired at rest and after CPT. Native myocardial T1 and T2 relaxation times, extracellular volume fraction (ECV), T2 signal intensity ratio, biventricular volumes and LGE were assessed in each patient.

**Results:**

SSc patients had significantly higher mean myocardial T1 (1029±32ms vs. 985±18ms, p<0.01), ECV (30.1±4.3% vs. 26.7±2.4%, p<0.05) and T2 (50.1±2.8ms vs. 47±1.5ms, p<0.01) values compared with controls. No significant differences were found between absolute MBF values at rest and after CPT; whereas lower MBF variation after CPT was observed in SSc patients (+33 ± 14% vs. +44 ± 12%, p<0.01). MBF variation had inverse correlation with native T1 values (r: -0.32, p<0.05), but not with ECV.

**Conclusions:**

Myocardial involvement in SSc at preclinical stage increases native T1, T2 and ECV values, reflecting inflammation and fibrosis, and reduces vasodilatory response to CPT, as expression of microvascular dysfunction.

## Introduction

Systemic sclerosis (SSc) is a multisystem connective tissue disease characterized by vasculopathy, immunologic disturbances and diffuse fibrosis of the skin and major organs.

Primary myocardial involvement is reported in around 14.5% of SSc patients, and higher prevalence is documented in autopsy studies [1, 2].

Although generally asymptomatic and clinically silent for years, cardiac involvement confers higher mortality risk, due to arrhythmia, systolic dysfunction and heart failure [3]. At histology, the hallmarks of SSc-related cardiomyopathy are patchy myocardial fibrous replacement, concentric intimal proliferation with fibrinoid necrosis of intramural arteries and contraction band necrosis, which is characteristic of reperfused tissue following transient vessel occlusion [1, 4].

Endothelial cell dysfunction appears to play a central role in the pathogenesis of SSc-related cardiomyopathy, leading progressively to microvascular obliteration, ischaemic damage and increased fibroblast activity [5].

Myocardial damage is supposed to be caused by multiple repeated focal ischaemic-reperfusion injuries, unrelated to coronary artery disease and resulting from coexistence of structural microvascular impairment and abnormal vasoreactivity [6].

Myocardial native T1 (nT1) values and extracellular volume fraction (ECV), as surrogates of interstitial expansion and fibrosis, may identify myocardial injury in SSc even in the early and subclinical stages of the disease [7–9], even when not detectable by conventional Cardiovascular Magnetic Resonance (CMR) techniques, such as T2-weighted sequences and late gadolinium enhanced (LGE) imaging. Likewise, T2 mapping technique has been demonstrated to be superior to conventional T2-weighted sequences in the detection of myocardial inflammation in rheumatic and autoimmune diseases [10].

The cold pressor test (CPT) enables in vivo assessment of the coronary endothelial function, since it induces vasodilatation of coronary resistance vessels by activating a combination of sympathetic adrenergic effects and the release of endothelium-derived relaxation factors [11].

The evaluation of hypothermic-induced physiologic changes in myocardial blood flow (MBF) can be used as measure of the integrity of endothelial function [11].

Our purpose was to investigate the presence of diffuse myocardial damage in asymptomatic SSc patients without known cardiac disease using CMR T1 and T2 mapping techniques and its correlation with cold-induced vasoreactivity, compared to a healthy age- and sex- matched control group.

## Materials and methods

### Study population

Forty consecutive SSc patients (mean age: 42.1 ± 11.2 years, 30 women) were prospectively enrolled by the Clinical Immunology Unit-Scleroderma Center at the Department of Clinical Medicine of Policlinico Umberto I in Rome.

All patients fulfilled the 2013 American College of Rheumatology/European League Against Rheumatism (ACR/EULAR) classification criteria for SSc, underwent routine clinical biohumoral assessment, echocardiography and CMR [12].

Exclusion criteria included cardiac symptoms (typical angina, palpitation and dyspnoea), history of ischemic or non-ischemic cardiac diseases, reduction of ventricular contractile function (ejection fraction < 45%), signs of pulmonary hypertension assessed by Doppler Echocardiography, renal failure (estimated glomerular filtration rate <30ml/min), pregnancy, known hypersensitivity to gadolinium or contraindication to CMR, age < 18 or > 59 years.

Subjects with history of diabetes, hypertension, hypercholesterolemia and smoking were excluded to minimize confounding factors that could determine microvascular or endothelial dysfunction.

Ten controls referring to our center to perform a contrast-enhanced magnetic resonance imaging (MRI) exam of other districts (adrenal glands, pancreas, kidney) were enrolled matching the study group for age and sex. All controls have no history of cardiovascular, rheumatological and metabolic diseases, no smoking, no under vasoactive therapy and normal ECG.

The local ethics committee provided approval for this investigation and informed consent was obtained from all patients and controls.

### Clinical assessment

An expert clinical immunologist performed physical examinations and structured interviews.

Skin thickening was assessed by the modified Rodnan Skin Score (mRSS), determined by a standardized pinching method at 17 different sites of the body.

According to Le Roy et al. [13], SSc patterns were classified as limited cutaneous SSc (lcSSc) or diffuse cutaneous SSc (dcSSc).

The SSc disease activity was evaluated using the Valentini disease activity index (DAI) proposed by the European Scleroderma Study Group in 2001 [14], which combines skin changes, pulmonary function test, erythrocyte sedimentation rate (ESR), scleroedema, digital necrosis, articular and muscular involvement, and hypocomplementaemia.

SSc disease severity was assessed by Medsger Disease Severity Scale (DSS), which scored multiple organ and system involvement separately from 0 to 4 (no, mild, moderate, severe or end-stage involvement).

Nailfold videocapillaroscopy was examined in each patient according to patterns proposed by Cutolo, including “early”, “active” and “late” [15].

At the time of enrolment, all SSc patients were undergoing treatment with calcium channel blockers (nifedipine 30 mg/day), prostanoid therapy and 16 Bosentan for digital ulcers prevention; however, these drug therapies have been suspended at least three days before performing CMR in order to not impair perfusion evaluation. None of the patients was treated with immunosuppressive agents.

### CMR acquisition protocol

CMR imaging was performed with a 1.5-T unit (Magnetom Avanto; Siemens Healthcare, Erlangen, Germany) using an 8-channel phased-array coil.

**Fig 1:**
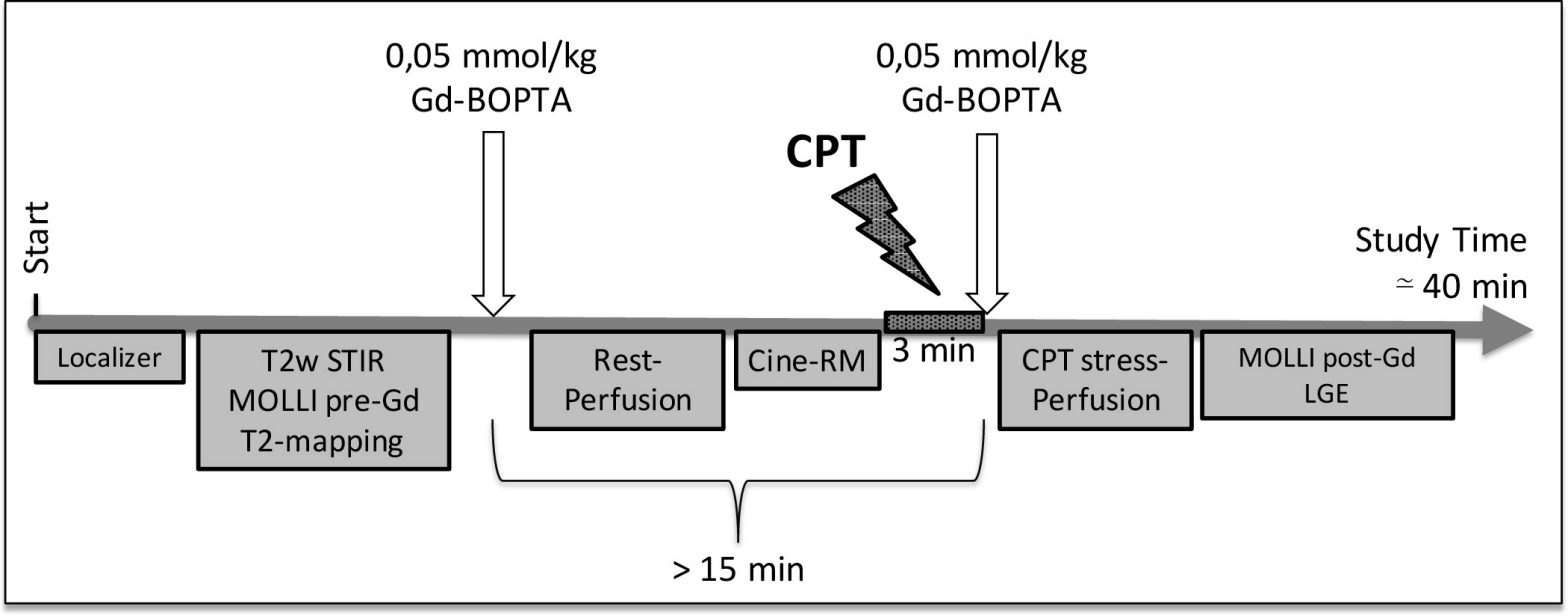
Comprehensive CMR protocol scheme. CPT = cold pressor test; Gd-BOPTA = gadobenate dimeglumine; T2w STIR = Short tau inversion recovery T2-weighted sequence; MOLLI = Modified Look-Locker Inversion recovery sequence; pre-/post-Gd = before/after gadolinium administration.

The comprehensive CMR protocol scheme is shown in Fig 1 and included:

- b-SSFP sequences (cineMR) were acquired with standard cardiac planes to assess biventricular function.
- T1 mapping was performed using the modified Look-Locker inversion recovery (MOLLI) sequence on three short-axis views (one basal and two mid-ventricular) before and 15 min after the second bolus injection of contrast agent at the identical slice location. Three contiguous slices have been acquired in basal and mid ventricle, where ventricular walls are thicker, in order to give a more representative and robust estimation of the T1 value and to avoid potential confounders, as blood partial voluming being a particular concern in apical segments. ECV value was determined according with the established delayed post-contrast bolus protocol.
- T2 map was obtained using a T2-prepared True-FISP prototype sequence producing 3 single shot images with 3 different T2 pulse preparation. Non-rigid registration algorithm and the two-parametric automatic curve fitting were automatically applied to generate the map [16].
- Focal myocardial edema areas were detected by acquiring short tau inversion recovery (T2w) sequences by activating the only body coil.
- LGE imaging was performed between 10 and 15 min after the second bolus injection of contrast agent using a segmented T1-weighted phase-sensitive inversion-recovery pulse.

For perfusion study, series of single-shot gradient-echo pulse sequence were acquired after injection of a double bolus of 0.05 mmol/kg of body weight of gadobenate dimeglumine (Gd-BOPTA, MultiHance, Bracco Imaging S.p.A., Milan, Italy) at 3 ml/s followed by a flush of 20 ml of saline solution at rest and after CPT, with a 15-minute interval between two administrations. Three basal and mid-ventricular short-axis sections with an interslice gap of 10mm were acquired at end-expiratory breath hold on midsystole, which represents the cycle phase in which the myocardial walls have the maximum thickness. Stress perfusion imaging was acquired immediately after the CPT, consisting in immerging completely both hands in two bowls containing ice water (0–4°C) positioned on the both side of the table for 180 seconds, once the patient table has been moved out the gantry, to create an adequate stimulus. Immediately at the end of the stimulus the patient is reintroduced into the gantry and one of the bowls positioned above the patient's head to continue the stimulus, while perfusion sequence and bolus contrast injection start.

Sequences details are reported on S1 Appendix.

### CMR image analysis

All CMR studies were analyzed using a dedicated software for advanced CMR image analysis (cvi42© vers. 5.3, Circle Cardiovascular Imaging, Calgary, Canada).

#### Function assessment

Left and right ventricular (LV and RV) volumes were calculated semiautomatically by contouring endocardial and epicardial borders on end-diastolic and end-systolic short-axis cineMR images and indexed for body surface area (BSA).

#### T2w and LGE images

T2 ratio was calculated by relating the average myocardial signal intensity of the entire left ventricle to that of the skeletal muscle, as reccomended in assessing inflammation to diagnose myocarditis [17]; myocardial edema was defined when myocardial T2 ratio was > 1.9. The same threshold was also employed in the detection of focal areas of edema.

Presence of focal fibrotic areas was assessed on LGE images as myocardial regions of increased signal intensity value 5 standard deviations (SDs) above the mean signal intensity of the remote normal myocardium, with a contiguous area of ≥ 40 mm^2^, to minimize cofounding effect of noise.

#### T1, T2 and ECV mapping

nT1 and postcontrast T1 maps, as well as ECV maps, were generated by using a dedicated software (Cmr42 v.5.3.0, Circle Cardiovascular Imaging Inc., Calgary, Alberta, CA).

Global myocardial nT1 and ECV values were assessed respectively on native and ECV maps, by averaging the global values obtained in the three maps, measured by contouring manually internally subepicardial and subendocardial layers and avoiding LGE areas as previously described [18].

T2 value was measured on the generated T2 map drawing a ROI within the interventricular septum, avoiding epicardial fat, ventricular cavity or misregistration artifacts.

#### Myocardial blood flow

Endocardial and epicardial contours and a ROI in the ventricular cavity were manually traced at the point of greatest left ventricular blood pool signal intensity. The contours were automatically propagated through to all time point and then manually corrected for breathing displacement and being careful to draw the lines in the myocardial wall. Whole myocardial and blood pool signal intensities were measured in each time point during and after contrast injection in each ventricular plane. Global MBF was defined as mean transmural flow of all myocardial segments.

MBF was calculated by using the Fermi-constrained de-convolution technique [19] implemented with the Matlab software platform (Version 7; The MathWorks, Natick, Mass), by converting signal intensity values into concentrations, using the signal intensity equation for the imaging pulse sequence as previously described [20]. Specific methods of this conversion are detailed in S2 Appendix.

The modification of the value of MBF after CPT was evaluated as follow:

$$\%\;MBF\;variation\;after\;CPT=\left[\left(MBF_{postCPT}-MBF_{preCPT}\right)/MBF_{preCPT}\right]\;*\;100$$

### Statistical analysis

Descriptive parametric values with a normal distribution are presented as mean +/- standard deviation (SD); normality was determined using the Shapiro-Wilks test. If highly skewed, parametric data were reported as median (interquartile range); nonparametric data are presented as frequences and percentages.

Dichotomous data have been compared using the chi-square test or Fischer's exact test.

Baseline demographic characteristics of the subjects were compared using unpaired t-test and Chi-square test for continuous variables and categorical variables, respectively. Between-subgroup differences in SSc and control were tested with unpaired t-tests. For dcSSc, lcSSc and control, we Bonferroni-corrected to type I error = 0.017(0.05/3). Bonferroni correction was likewise used for multiple correlations (refer to respective results tables). Bootstrap corrections were applied to correct for potential outliers for significant correlations found. Bivariate correlations were assessed using Pearson’s or Spearman’s or Kendall's tau coefficient, as appropriate. Multivariate stepwise regression analyses with several potentially confounding factors were performed with nT1, ECV and MBF as the dependent variable. P-value of less than 0.05 was considered to indicate a statistically significant difference. The 95th percentile values of control group were calculated and used as threshold values to define the abnormal increase of nT1, ECV and T2 and the abnormal variation of MBF after CPT in SSc patients. Statistical analysis was performed using the software package SPSS (version 20.0; IBM SPSS, Chicago, Ill).”

## Results

### Clinical features

In SSc patient group, the duration of disease was 8.7 ± 7.3 years, the DAI, the DSS and the mRSS were respectively 1.6 ± 1.4, 3.3± 2 and 13.6 ± 6.5 (mean ± SD), indicating a low grade of active disease and a moderate organ involvement. All patients presented Raynoud’ phenomenon. Overall clinical features are summarized in Table 1.

**Table 1:** SSc patient group: Clinical characteristics.

SSc patient group: clinical features	
Clinical and demographic features	
Age (ys, mean ± SD)	42.1 ± 11.2
Sex (male, %)	10 (25)
dcSSc vs lcSSc (n, %)	25 / 15 (63 / 37)
Disease activity and chronicity indices	
Disease duration (ys, mean ± SD)	8.7 ± 7.3
Duration from onset of Raynoud’ phenomenon (ys, mean ± SD)	10.2 ± 8.7
Valentini Disease Activity Index (mean ± SD)	1.6 ± 1.4
Disease Severity Score (mean ± SD)	3.3 ± 2.0
Modified Rodnan Skin Score (mean ± SD)	13.6 ± 6.5
Erythrocyte sedimentation rate (mm/h, mean ± SD)	24.4 ± 17.0
DLCO (mean ± SD)	76.9 ± 10.9
DLCO ≥ 70 (n, %)	30 (75)
Interstitial lung disease (n, %)	17 (42.5)
Capillaroscopy Early/Active/Late (n, %)	11 / 12 / 17 (27 / 30 / 43)
Myositis (n, %)	4 (10)
Digital Ulcers (n, %)	26 (65)
Renal crisis (n, %)	0 (0)
SSc-specific autoantibodies,	
Patients with Anti-topoisomerase I antibodies (n, %)	23 (57.5)
Patients with Anticentromere antibodies (n, %)	9 (22.5)
Patients with no Scl-70 and no ACA antibodies (n, %)	8 (20)

Continuous variables are expressed as mean value ± standard deviation (SD), while categorial variables are expressed as frequency and percentage; BMI, body mass index; dcSS, diffuse systemic sclerosis; lcSSC, limited cutaneous systemic sclerosis; DLCO: carbon monoxide diffusing capacity; Scl-70, Anti-topoisomerase I; ACA, anticentromere.

### Ventricular function

All patients and controls well tolerated CMR and CPT without onset of any cardiac symptoms or other complications.

No significant differences were emerged in LV volumes, mass and ejection fraction between SSc patients and controls. A slight, non-statistically significant, enlargement of RV-ESV and reduction of RV-EF were observed in the SSc cohort (Table 2).

**Table 2:** SSc patient group: CMR features.

Cardiac Magnetic Resonance Features	SSc Patients (n = 40)	Controls (n = 10)	*p value*
**Ventricular Function**			
LV-end diastolic volume (mL/m^2^; mean ± SD)	71.7 ± 11.8	75.7 ± 14.1	*0*.*357*
LV-end systolic volume (mL/m^2^; mean ± SD)	28.0 ± 6.2	31.2 ± 6.5	*0*.*151*
LV-stroke volume (mL/m^2^; mean ± SD)	43.4 ± 7.5	44.4 ± 9.1	*0*.*705*
LV-ejection fraction (%; mean ± SD)	60.9 ± 5.4	58.6 ± 4.1	*0*.*213*
LV-Mass (g/m^2^; mean ± SD)	45.2 ± 9.4	44.0 ± 9.8	*0*.*722*
RV-end diastolic volume (mL/m^2^; mean ± SD)	76.1 ± 14.6	68.4 ± 11.4	*0*.*129*
RV-end systolic volume (mL/m^2^; mean ± SD)	33.6 ± 10.8	25.3 ± 7.2	*0*.026*
RV-stroke volume (mL/m^2^; mean ± SD)	42 ± 7.5	43.0 ± 8.1	*0*.*725*
RV-ejection fraction (%; mean ± SD)	56.5 ± 7.1	62.8 ± 7.5	*0*.015*
**Tissue characterization**			
LGE (n, %)	12 (30)	0 (0)	*0*.012*
Focal oedema (n, %)	5 (12)	0 (0)	*0*.*239*
Pericardial effusion (n, %)	7 (23)	1 (10)	*0*.021*
Global T2 SI Ratio (mean ± SD)	1.61 ± 0.3	1.45 ± 0.3	*0*.*200*
T2 (ms; mean ± SD)	50.1 ± 2.8	47.0 ± 1.5	*0*.002**
T2 > 49.7ms (n, %)	21 (52)	1 (10)	*0*.009**
nT1 (ms; mean ± SD)	1029.2 ± 32.1	984.9± 17.8	*< 0*.001**
nT1 > 1010 ms (n, %)	29 (72)	1 (10)	*< 0*.001**
Hematocrit (%, mean ± SD)	40.3 ± 3.3	42.3 ± 3.5	*0*.*255*
ECV (%; mean ± SD)	30.1 ± 4.3	26.9 ± 2.4	*0*.003**
ECV > 29.8% (n, %)	21 (52)	1 (10)	*0*.009**
**Perfusion indices**			
MBF at rest (ml/s, mean ± SD)	0.93 ± 0.06	0.89 ± 0.07	*0*.*111*
MBF after CPT (ml/s, mean ± SD)	1.23 ± 0.11	1.29 ± 0.07	*0*.*118*
% MBF variation after CPT (mean ± SD)	+33% ± 14%	+44% ± 11%	*0*.020*
% MBF increase after CPT < 33.8%	20 (50)	0 (0)	*0*.014*

Continuous variables are expressed as mean value ± standard deviation (SD), while categorial variables are expressed as frequency and percentage; LV, left ventricle; RV, right ventricle; LGE, late gadolinium enhancement; SI, signal intensity; nT1, native T1 value; ECV, extracellular volume fraction; CPT, cold pressor test; MBF, myocardial blood flow;

* p<0.05;

** p<0.01.

### T2w and LGE images

**Fig 2:**
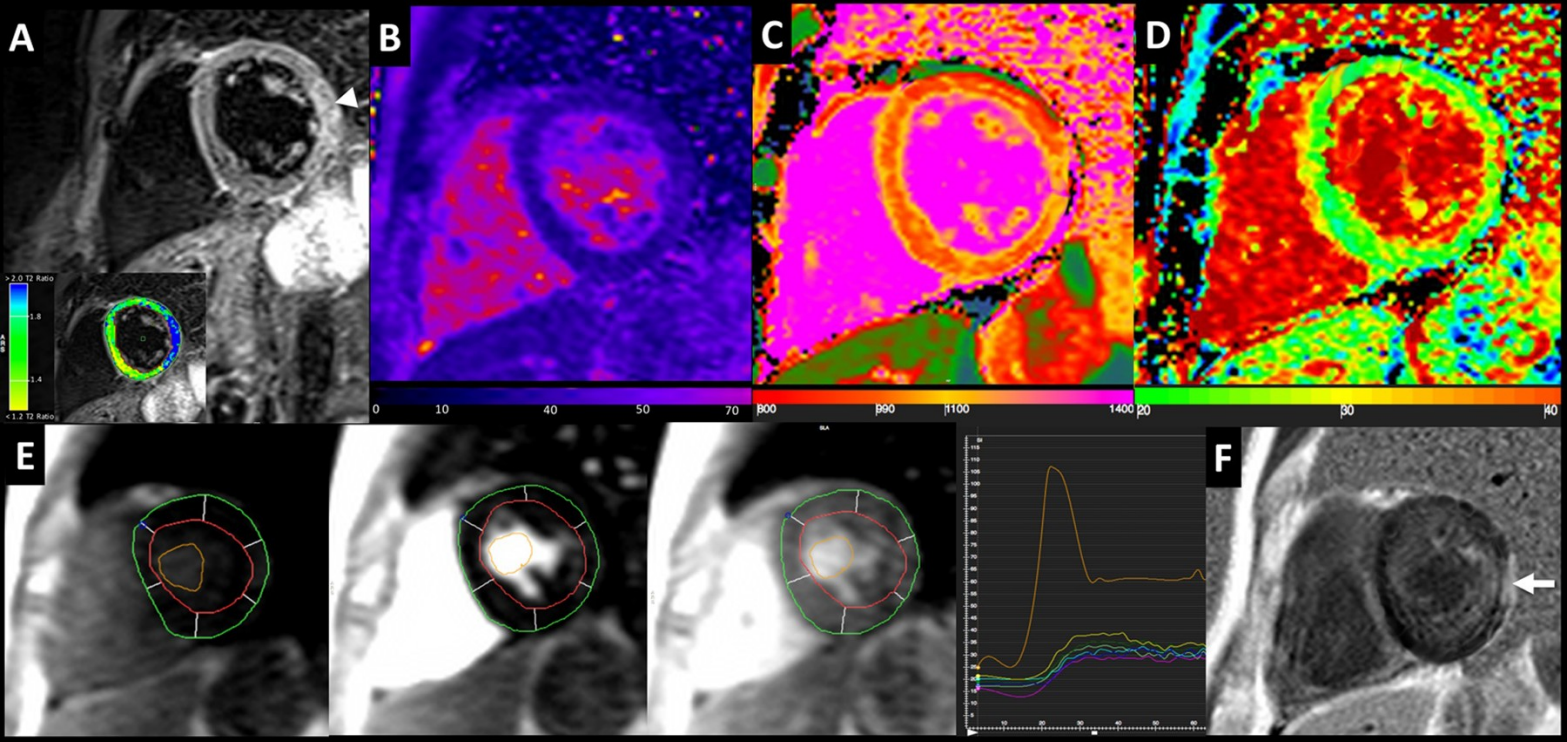
40 years-old male SSc patient (dcSSc pattern) with 13-years history of SSc disease duration. STIR-T2w image shows an area of myocardial edema on left ventricular lateral wall (arrow), emphasized by the analysis of T2 myocardial-to-skeletal muscle signal intensity ratio (box below). Analysis of myocardial T2 (b.), nT1 (c.) and ECV (d.) maps showed T2 value of 51 ± 2.5 ms, a mild diffuse increase of global myocardial nT1 (1050 ± 24 ms) and a slight increase of ECV (33 ± 3.4%). Perfusion images (e.) before, during and after the passage of contrast bolus with relative signal intensity-time curves at different time-points of the myocardial segments and ventricular cavity. LGE image (f.) show a mid-subepicardial enhancement on the lateral wall (arrow).

On T2w imaging, SSc group showed areas of focal edema in 12% of patients and a mean T2 ratio of 1.61 ± 0.3. In 30% of SSc patients’ focal areas of LGE were found, all with nonischemic pattern (Fig 2), predominantly with patchy mesocardial distribution in the basal lateral wall (n = 8) or in the basal inferior septum (n = 4). No hyperintense areas in T2w and LGE images were found in controls.

### nT1, T2 and ECV values

**Fig 3:**
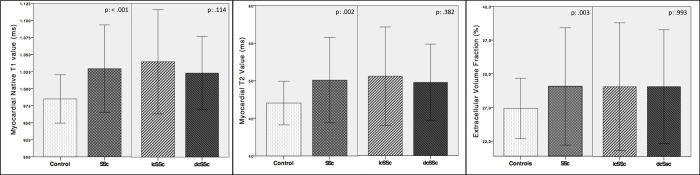
Box plot graphs of native T1, T2 and ECV. Mean values, +/- 1 Standard Deviation bars; SSc = systemic sclerosis; lcSSc = limited cutaneous form; dcSSc = diffuse cutaneous form.

SSc patients had significant higher native myocardial nT1 and T2 values compared to controls (SSc vs. controls: nT1 = 1029.2 ± 32.1 ms vs. 984.9 ± 17.8 ms, p < 0.01; T2 = 50.1 ± 2.8 ms vs. 47 ± 1.5 ms, p < 0.01; Fig 3A and 3B), also when considering the two distinct patterns of the disease (dcSSc/lcSSc Vs. controls, p < 0.01, Table 3).

**Table 3:** Clinical characteristics and CMR features of dcSSc and lcSSc patient subgroups and paired comparison.

	dcSSc Patients (n = 25)	*p value dcSSc vs controls*	lcSSc Patients (n = 15)	*p value lcSSc vs controls*	*p value dcSSc vs lcSSc*
**Clinical Features**					
Age (ys, mean ± SD)	38.9 ± 10.1	*0*.*10*	47.5 ± 11.2	*0*.*19*	*0*.02*
Disease duration (ys, mean ± SD)	6.8 ± 5.4	-	11.7 ± 9.2	-	*0*.04*
Duration from onset of RP (ys, mean ± SD)	8.6 ± 7.2	-	12.7 ± 10.3	-	*0*.*15*
DAI (mean ± SD)	2.1 ± 1.5	-	0.9 ± 0.9	-	0.008**
DSS (mean ± SD)	3.8 ± 2.2	-	2.4 ± 1.2	-	*0*.03*
**Ventricular Function**					
LV-end diastolic volume (mL/m^2^; mean ± SD)	72.1± 11.4	*0*.*45*	70.9 ± 12.8	*0*.*39*	*0*.*77*
LV-end systolic volume (mL/m^2^; mean ± SD)	28.4 ± 4.9	*0*.*19*	27.2 ± 7.9	*0*.*20*	*0*.*54*
LV-stroke volume (mL/m^2^; mean ± SD)	43.7 ± 7.9	*0*.*82*	42.8 ± 7.1	*0*.*63*	*0*.*73*
LV-ejection fraction (%; mean ± SD)	60.5 ± 4.2	*0*.*23*	61.6 ± 7.1	*0*.*24*	*0*.*55*
LV-Mass (g/m^2^; mean ± SD)	44.1 ± 8.9	*0*.*98*	47.1 ± 10.3	*0*.*47*	*0*.*34*
RV-end diastolic volume (mL/m^2^; mean ± SD)	76.3 ± 16.2	*0*.*17*	75.7 ± 12.1	*0*.*14*	*0*.*89*
RV-end systolic volume (mL/m^2^; mean ± SD)	32.9 ± 11.5	*0*.*06*	34.8 ± 9.6	*0*.014*	*0*.*58*
RV-stroke volume (mL/m^2^; mean ± SD)	43.4 ± 7.6	*0*.*86*	39.6 ± 6.8	*0*.*27*	*0*.*11*
RV-ejection fraction (%; mean ± SD)	57.7 ± 6.9	*0*.*06*	54.5 ± 7.1	*0*.009**	*0*.*16*
**Tissue characterization**					
Global myocardial T2 SI Ratio	*1*.*54 ± 0*.*3*	*0*.*48*	*1*.*72 ± 0*.*4*	*0*.*09*	*0*.*13*
T2 (ms; mean ± SD)	49.8 ± 2.5	*0*.003**	50.6 ± 3.3	*0*.004**	*0*.*38*
T2 > 49.7ms (n, %)	11 (45)	*0*.040*	10 (60)	*0*.005**	*0*.*16*
nT1 (ms; mean ± SD)	1023 ± 27	*0*.000**	1040 ± 38	*0*.000**	*0*.*11*
nT1 > 1010ms (n, %)	18 (72)	*0*.001**	11 (73)	*0*.002**	*0*.*93*
ECV (%; mean ± SD)	30.1 ± 4.2	*0*.006**	30.1 ± 4.7	*0*.031*	*0*.*99*
ECV > 29.8% (n, %)	12 (48)	*0*.036*	9 (60)	*0*.012*	*0*.*46*
**Perfusion indices**					
MBF at rest (ml/s, mean ± SD)	0.91 ± 0.06	*0*.*37*	0.94 ± 0.05	*0*.037*	*0*.*10*
MBF after CPT (ml/s, mean ± SD)	1.23 ± 0.10	*0*.*16*	1.21 ± 0.11	*0*.*11*	*0*.*65*
% MBF variation after CPT (mean ± SD)	+35.2 ± 12	*0*.039*	+29.7 ± 16	*0*.017*	*0*.*21*
% MBF increase after CPT < 33%	11 (44)	*0*.040*	9 (60)	*0*.012**	*0*.*327*

Continuous variables are expressed as mean value ± standard deviation (SD), while categorial variables are expressed as frequency and percentage; dcSSc, diffuse systemic sclerosis; lcSSc, limited systemic sclerosis; RP, Raynaud’ Phenomenon; DAI, Disease activity index; DSS, disesase severity scale; LV, left ventricle; RV, right ventricle; SI, signal intensity; nT1, native T1 value; ECV, Extracellular Volume Fraction; CPT, cold pressor test; MBF: myocardial blood flow.

*p<0.05 not significant after Bonferroni correction, and

e.g. <0.017** significant after Bonferroni correction for family-wise error for each subgroup comparison

SSc patients showed a significantly higher ECV (30.1 ± 4.3%) than controls (26.7 ± 2.4%, p = .049, Fig 3C), whereas there were no differences in myocardial ECV between patients with and without LGE. A slight non-significant increase of nT1 and T2 values was noted in lcSSc subgroup compared to dcSSc. Ninety-fifth percentile values of nT1, T2 and ECV measurements in healthy controls were respectively 1010ms, 49.7ms and 29.8%.

### Myocardial blood flow

At rest, MBF was slightly higher in the SSc patients (0.93 ± 0.06 mL/g/min) compared to control group (0.89 ± 0.07 mL/g/min, *p*:*0*.*13*); MBF values measured after CPT did not show significant differences between two groups (SSc group vs. controls: 1.29 ± 0.07 vs 1.23 ± 0.11 mL/g/min).

**Fig 4:**
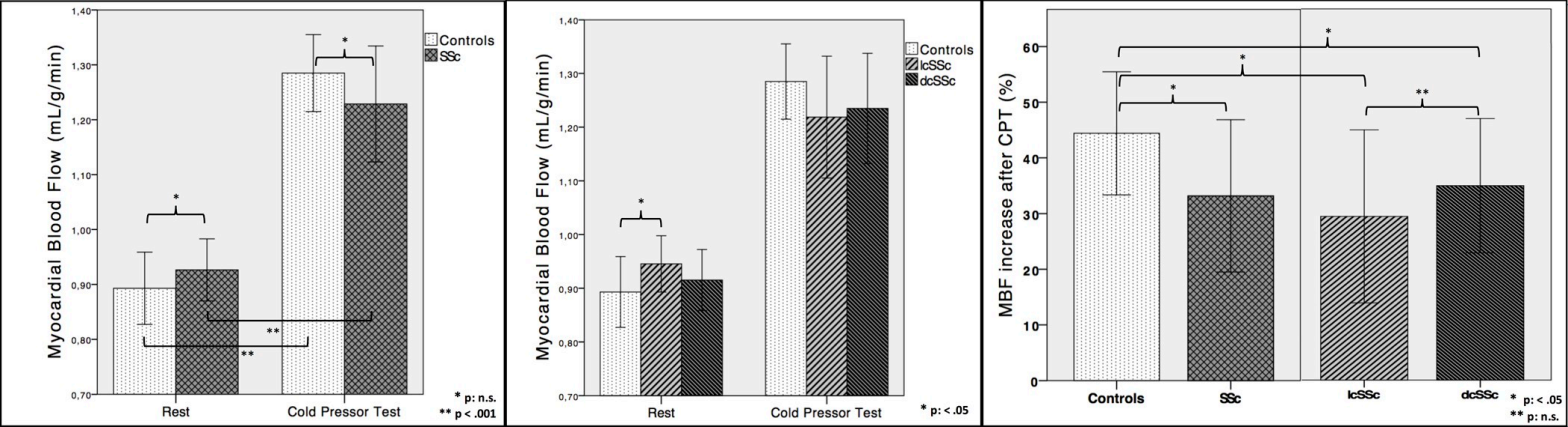
Box plot graphs of myocardial blood flow (MBF) at rest and after cold pressor test (CPT). Mean values, +/- 1 Standard Deviation bars; SSc = systemic sclerosis; lcSSc = limited cutaneous form; dcSSc = diffuse cutaneous form.

MBF increase in response to hypothermic stimulus was lower in SSc (33.1 ± 14%) compared to controls (44.2 ± 12%, *p* = .022), but did not show any difference between subgroups (diffuse vs. limited SSc forms p: .227, Fig 4). Lower 95th percentile values of % MBF variation after CPT in healthy controls was 33%, and this value was used to detect abnormal vasodilation response to CPT.

### Correlation of myocardial nT1, T2 and ECV to MBF

**Fig 5:**
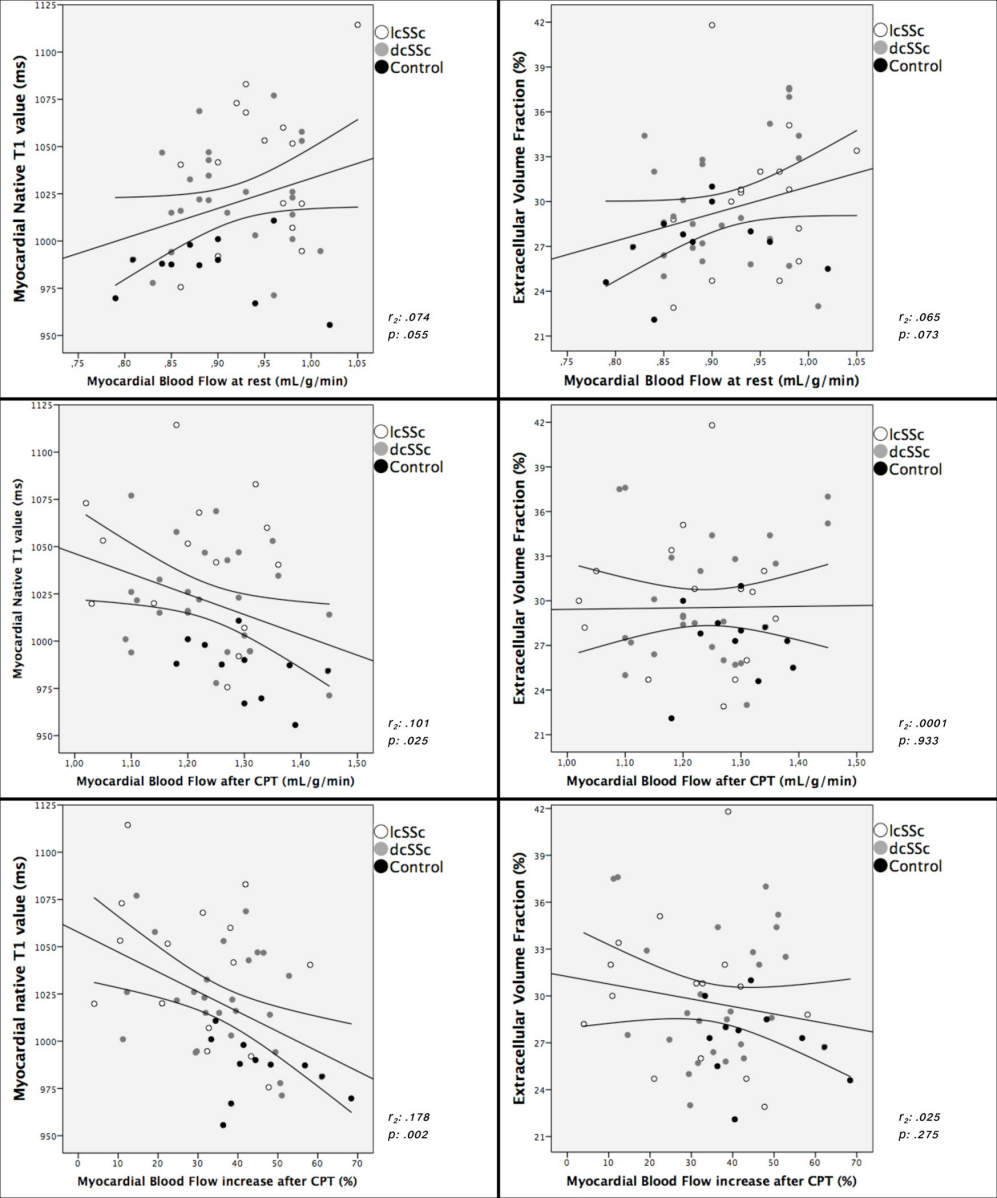
Myocardial nT1 and ECV plotted against MBF at rest, after cold pressor test (CPT) and %MBF variation after CPT.

nT1 and ECV values were positively correlated (p: .013) and both had a strong correlation with T2 values (p < .01 for both). Interestingly, an inverse relationship between nT1 value and MBF increase after CPT was also found (r: -0.42, p < .002), suggesting a link between myocardial tissue involvement and vasoreactivity, whereas ECV did not correlate with the response to hypothermic stimulus (r: -0.16, p: .275, Fig 5). Furthermore an inverse association between nT1 and MBF after CPT (r: -0.32, p: .025) and a trend of direct correlation with rest MBF (r: 0.27, p: .055) were noted.

Moderate positive correlation was noted between nT1 vs. mRSS and VES, ECV vs. age, disease activity index and disease duration (p < .05 for all).

MBF after CPT and MBF variation after CPT had inverse relationships with disease duration (both from time of onset of disease and from Raynaud’s phenomenon); those values were positively correlated with MBF at rest (p < .05 for all).

nT1, T2, ECV and MBF variables were then entered into a backward stepwise multivariate logistic regression model (Table 4). Multivariate logistic regression revealed that nT1 and ECV remained a significant and independent predictor of SSc (OR 9.9, 95% CI 1.15 to 86; p = 0.037, OR 16, 85% CI 2.8 to 90).

**Table 4:** Logistic Regression Analysis for the significant and independent predictors in patients with SSc.

Group	Parameters	OR	Lower	Upper	*p-value*
SSc vs Control	**MBF**	5.4	0.3	103	*0*.*255*
	**ECV**	9.9	1.15	86	*0*.037*
	**nT1**	16	2.8	90	*<0*.001*
	**nT2**	11	1.27	95.1	***0*.*029***
dcSSc vs lsSSc	**MBF**	0.4	0.01	2.1	*0*.*301*
	**ECV**	0.4	0.1	1.5	*0*.*169*
	**nT1**	1.0	0.2	4.9	*1*.*000*
	**nT2**	0.46	0.12	1.7	*0*.*254*
dcSSc vs Control	**MBF**	4.3	0.2	89	*0*.*336*
	**ECV**	7	0.8	64.6	*0*.*083*
	**nT1**	16	2.5	100	*0*.003*
	**nT2**	8.3	0.91	75.7	***0*.*060***
lsSScvs Control	**MBF**	8.2	0.4	171	*0*.*174*
	**ECV**	18	1.7	184	*0*.015*
	**nT1**	8	1.2	52	*0*.031*
	**nT2**	18	1.75	184	***0*.*015***

OR: Odds ratio, CI: confidence Interval.

## Discussion

To the best of our knowledge, this is the first study that simultaneously demonstrated endothelial dysfunction of coronary microcirculation and the subclinical structural myocardial injury in young asymptomatic SSc patients with preserved ventricular function.

In this study, SSc-related myocardial involvement at preclinical stage was revelead by enlongation of myocardial nT1 and T2 values, expansion of ECV, and alteration of microvascular endothelium-dependent vasomotility evoked by CPT.

### Inflammation and fibrosis

In our patient’s cohort, areas of focal fibrosis on LGE and myocardial edema on T2w images were identified respectively in 30% and 12% of cases.

Previous studies report a large heterogeneity of prevalence rate of LGE areas in SSc ranging from 15% to 66% [21–25], reflecting the miscellaneous patient cohorts enrolled. Conversely, the use of T2w sequences in SSc has been rarely investigated [8, 23, 26] and the prevalence rates reported (around 12–13%) were not substantially different from ours.

In this study, SSc patients presented higher global nT1, T2 value and ECV compared to controls, even in absence of overt ventricular contractile dysfunction.

The higher nT1 value likely corresponds to nonspecific increase of tissue water content commonly related to diseases with acute presentation, chronic injury and other conditions causing interstitial expansion (e.g. myocardial fibrosis) or swelling of cardiomyocytes [27].

A large number of SSc patients resulted with global increase of nT1 and T2 values (respectively 72% and 52%). A high direct correlation was noted between nT1 and T2 values, as these CMR features are both altered by an increase of extra- and/or intracellular free fluid content.

ECV has been histologically validated as the best non-invasive surrogate of myocardial interstitial fibrosis [28] and is associated to poor outcomes [29]. In our cohort, more than half of SSc patients (52%) showed increased in ECV, which was correlated with both nT1 and T2.

We found a different prevalence of patients with isolated nT1 elongation (30%, in most cases with concomitant increase in the T2 value) and combined nT1/ECV increase (42.5%). These tissue signal abnormalities could express a combination of low grade inflammation (increase of nT1 and T2) and diffuse myocardial fibrosis (expanded ECV) [8, 30], that might precede the contractile impairment occurring at late stage of SSc-related cardiopathy[30].

Few studies evaluated the myocardial remodelling in SSc patients, using T1 mapping technique [7–9, 25, 31].

Thuny et al. [7] found a significant increase of ECV in SSc patients with normal LV function and no LGE, which correlated with a reduction of circumferential strain. Ntusi et al. [8] also noted expanded ECV, which was accompanied by higher nT1 values. In contrast to those results, in our subgroup analysis no significant differences are evident in CMR tissue features between lcSSc and dcSSc patients, even though the latter had slightly lower nT1 than the former, which could be also influenced by the differences in age and duration of disease in the composition of the two groups.

Barison et al. [9] found an expansion of ECV in 30 a-/paucisymptomatic patients (93% presenting lcSSc form) with short disease duration (median = 2.5 years), but no significant abnormalities in nT1 values. This discrepancy with our results could be attributable also to different MR system and T1 mapping sequence employed (modified-cine inversion-recovery).

In addition, it has been demonstrated that nT1 and ECV correlate with an increase in circulating SSc activity markers (growth differential factor 15) and predictor of heart failure development (Galectin-3) [25].

Cardiac involvement in progressive SSc may remain clinically silent for years and it is associated with a poor prognosis when first clinical signs become apparent, as documented by an event rate of 28% (including cardiovascular death, shock, arrhythmic event, and heart failure-related re-hospitalization) at 22.5 months follow-up [32].

The detection of abnormal myocardial CMR tissue features might play a crucial role in the cardiovascular risk stratification of SSc patients, selecting those who can benefit from specific treatments that could prevent the progression of cardiovascular injury (e.g. anti-inflammatory or vasoactive therapies) or a closer follow-up scheme.

The elevation of at least one value between nT1, T2 and ECV or detection of myocardial edema or LGE areas could be sufficient to identify early subclinical stage of SSc-related myocardial involvement [30].

In fact, although the presence of myocardial edema at CMR could be considered as an early hallmark of myocardial involvement, T2 mapping technique may underestimate the low-grade edema in spite of giving true T2 values and T1 mapping suffers from lower specificity due to changes reported in many diseases with higher extracellular compartment [33].

Moreover, there is a large overlap in mapping values between SSc patients and healthy subjects, therefore to define the limit between normal and pathological may be difficult.

In our study, we proposed the upper limit of the normal range (95th percentile) as the threshold for mapping values and we found at least one abnormal value in the vast majority of patient population in our cohort (85%), which could demonstrate how predominant and underestimated is the myocardial involvement at the pre-clinical phase.

However, clinical and prognostic implications of early SSc-related myocardial involvement still need largely to be clarified with further studies, possibly with long follow-up.

### Myocardial perfusion and endothelial function

The increase of global MBF after CPT observed in all subjects proved the effective coronary vasodilation evoked by cold stimulus. However, despite the similar hemodynamic response to CPT between patients and controls, the increase of MBF was significantly less pronounced in SSc group: such difference could represent an impairment of the endothelium-dependent microvascular function occurring in subclinical stages of SSc.

Myocardial microvascular dysfunction (MD) in SSc has been rarely investigated. A decreased myocardial perfusion was documented in SSc patients with no coronary disease during adenosine stress [34], as indirectly assessed by measuring coronary sinus flow using velocity-encoded sequence.

A significant reduction of myocardial perfusion reserve index at perfusion CMR was observed in a small number of subjects with Raynaud's phenomenon (included 9 SSc patients) [35] compared to healthy controls.

Mizuno et al [36] demonstrated a reduction of MBF assessed by contrast echocardiography 4 minutes after cold provocation, in 29.4% of SSc asymptomatic patients.

Gustafsson et al. [37] found cold-induced reversible perfusion defects in 12/21 patients using SPECT.

In our cohort, nearly half of the SSc patients (60% of lcSSc and 44% of dcSSc patients) had a reduced increase of MBF after CPT, which likely reflects endothelial function impairment.

Interestingly, this patient subgroup had higher nT1 and ECV, that could confirm the theorized parallelism between microvascular disease severity and progression of myocardial SSc-related injury [5].

The hypothesis that a dysregulation of coronary microcirculatory dynamics, the so-called “cardiac Raynaud’s phenomenon”, is involved in the determination of the cardiac damage is not yet definitively clarified [4].

Some authors speculated that repeated transient ischemia mediated by arteriolar vasospasms in response to various stimuli (including cold) might cause myocardial damage in SSc [36, 38].

Subclinical ischemic condition in SSc could be triggered by maladaptative mechanisms (reduction of the arteriolar bed’s cross-section due to obliterative microangiopathy, immune-mediated endothelial damage, defects of progenitor endothelial cells), leading to imbalance between vasoconstrictor and vasodilator mediators and consequently incongruous microvascular response to increased tissue blood flow demand [39].

A direct correlation between renal microvascular dysfunction, measured as increase of renal resistive index on by Doppler, and the occurrence of myocardial fibrosis detected as LGE areas has been recently demonstrated [40].

Moreover, the inverse correlation found in our results between the nT1 and the cold-induced MBF acceleration confirms the known association between MD and inflammation activity in patients with rheumatic diseases without obstructive coronary artery disease [41]. SSc pro-inflammatory molecules promote endothelial dysfunction by reducing the nitric oxide synthesis with consequent reduction of vasodilatatory modulation and increase of vascular adhesion of circulating inflammatory cells [41].

Finally, we have not found any significant difference in myocardial mapping, MBF values or CPT-induced vasoreactivity between lcSSc and dcSSc subgroups, whereas in other studies a higher incidence of MD was noted in the dcSSc [42]. Furthermore, the correlation that emerged between the nT1 and MBF values was not confirmed in the pattern subgroups, probably because their small dimension.

### Limitations

There are several limitations in this study. The small study population, therefore further studies with larger populations could reveal differences between subgroups. nT1 and ECV values may be altered in several pathological conditions, thus concomitant presence of other causes of myocardial inflammation or fibrosis cannot be fully excludable. No other serum or histological tests have been performed to support the evidence of diffuse myocardial fibrosis in our cohort; neither myocardial biopsy for histological correlations could be justified. Since all of our patients present the Raynaud phenomenon, it cannot be inferred that these evidences can be extended to patients without it.

## Conclusion

Subclinical myocardial involvement is common in asymptomatic SSc patients, as revealed by increase of nT1, T2 and ECV values, likely reflecting a combination of myocardial inflammation and diffuse fibrosis.

Reduced CPT-induced vasoreactivity in SSc may be considered an early expression of endothelial dysfunction, which might represent a precocious sign of microvascular impairment.

CMR could detect early signs of endothelial disfunction and subclinical myocardial involvement in SSc, improving prognostic stratification and addressing tailored therapy in order to prevent disease progression.

## Supporting information

S1 Appendix Detailed CMR protocol. (DOCX)

S2 Appendix Methods for conversion of perfusion data and calculation of MBF. (DOCX)

S1 Database Clinical and CMR database (patients and controls). (XLS)
