# Supplementary material for: Early myocardial damage and microvascular dysfunction in asymptomatic patients with systemic sclerosis: A cardiovascular magnetic resonance study with cold pressor test
Source: PLoS One. 2020 Dec 22;15(12):e0244282. doi: 10.1371/journal.pone.0244282 (PMC7755221; doi:10.1371/journal.pone.0244282)
Supplement: S1 Appendix — (DOCX) [file pone.0244282.s001.docx]

Appendix A

CMR sequence parameters and image reconstruction

- ***CineMR balanced-Steady state free-precession sequence***: TR 51,3, TE 1,21, a flip angle 45°, a 8 mm slice thickness, a matrix of 256x256, a field of view ranging from 340 mm to 400 mm and a voxel size of 2,0 x 1,3 x 8,0 mm. A stack of 10-12 contiguous short-axis slices completely encompassing both ventricles from the base to the apex was acquired.
- ***Modified Look-Locker inversion recovery prototype sequence***(package Siemens WIP #448) was acquired with following parameters: matrix 218 x 256, voxel size 1.41 x 1.41 x 8 mm3, TR/TE 2.6/1.12 ms, FA 35. For pre-contrast acquisitions the inversion pulse scheme was 5(3)3 consisting of 2 inversions with 5 images after the first inversion, a 3-heartbeat pause and then the last 3 images. For post-contrast acquisitions, the protocol was 4(1)3(1)2 consisting of 4 images acquired after the first inversion pulse and a one-heartbeat pause for the com- plete recovery of magnetization. Then 3 and 2 images, respectively, were acquired after the second and third inversion, separated by a one-heartbeat pause. Acquired images were then processed applying a motion correction algorithm provided in the package, designed to adjust in-plane mis-registration between images that may be caused by diaphragm position drift or heart beat irregularities. Two different sequence schemes were used for pre- and post-contrast acquisitions.

Extracellular volume fraction (ECV) map was than automatically generated by combining native and contrast-enhanced T1 map, insering hematocrit value sampled before starting CMR exam, using the following formula:

*ECV = (1 - hematocrit) x (ΔR1_tissue_ / ΔR1_blood_)*

where R1 = 1/T1 and the T1 values are given in milliseconds.

To measure global myocardial T1 value, epicardial and endocardial contours were manually drawn in native and contrast-enhanced images and aligned with the contours in each respective component image.

- ***T2-prepared True-FISP prototype sequence*** generated three different images with three different interval from T2 preparation pulse: 0ms, 24ms, and 55 ms (package Siemens WIP #448). Similarly to T1 mapping, acquired images were processed applying a motion correction algorithm.
- ***Short tau inversion recovery T2 weighted sequence (T2w-STIR)*** is a breath-hold black-blood segmented turbo spin echo sequence using a triple inversion recovery preparation module. Sequence parameters were: TR 2 R-to-R intervals, TE 75 ms, flip angle 180°, TI 170 ms, slice thickness 8 mm, no interslice gap, field of view 340 to 400 mm, matrix 256 x 256, and a voxel size of 2.3 x 1.3 x 8 mm).
- ***T1-weighted phase-sensitive inversion-recovery sequence*** for late enhancement sequence was setted with the acquisition window to mid-end diastole and the following parameters: field-of-view: 380 – 400 mm, slice thickness: 8 mm, repetition/echo time: 4.6/1.3 ms, flip angle: 208, matrix: 256 × 192. Meticulous attention was paid to select the inversion time to suppress the signal of normal myocardium with typical values of 250–320 milliseconds.
- ***Saturation-recovery single-shot gradient-echo perfusion pulse sequence*** parameters: repetition time msec/echo time msec, 2.7/1.0; preparation pulse delay (to the center of k-space), 150 msec; linear k- space order, shot duration 135 msec; flip angle, 15°; typical field of view, 380 mm 3 380 mm; two-fold sensitivity encoding; spatial resolution, 2.4 3 2.4 mm; and section thickness, 10 mm.
