# Supplementary material for: Early myocardial damage and microvascular dysfunction in asymptomatic patients with systemic sclerosis: A cardiovascular magnetic resonance study with cold pressor test
Source: PLoS One. 2020 Dec 22;15(12):e0244282. doi: 10.1371/journal.pone.0244282 (PMC7755221; doi:10.1371/journal.pone.0244282)
Supplement: S2 Appendix — (DOCX) [file pone.0244282.s002.docx]

Appendix B

Myocardial Blood Flow Analysis: the Deconvolution Model

Introduction

The Myocardial Blood Flow (MBF) was quantified from the blood pool and myocardial signal intensity versus time curves using a response function constrained deconvolution method [1], describes as follows. The amount of Contrast Agent (CA) in a stationary, linear system, represented by the tissue curve C_m_(t), can be related to the tracer concentration at the inlet, the Input Function (IF), represented by the blood curve C_b_(t), convolved with the Impulse Response Function (IRF), described by the formula

$C_{m}\left( t \right)=IRF\left( t \right)*C_{b}\left( t \right)=F*\left( R\left( t \right)*C_{b}\left( t \right) \right)$ (1)

where IRF(t) = F·R(t), R(t) is the response function, which represents the fraction of tracer that remains in the myocardium at time t and F is the rate of flow. Starting from Fick's mass balance equation [2] it is straightforward to derive an expression that relates the tracer amount in the region, C(t), to its input, in the form of a convolution integral. In according to Fick's principle (in integral form), also has to be equal to the amount of tracer that has entered the region, minus the amount that exited

$C\left( t \right)=\int c_{\mathrm{in}}\left( \tau-t \right)\cdot IRF\left( \tau\right)d\tau=F\int c_{\mathrm{in}}\left( \tau\right)-c_{\mathrm{out}}(\tau)d\tau$ (2)

The member on the right side of equation 2 follows from replacing the impulse function with a "Dirac-delta" input function. We also note, that with such an impulse input IRF (τ) = δ (t-τ) at time t = 0, there can be no tracer at the output c_out_(t = 0) = 0, as this would otherwise require that the tracer or contrast to pass through the region instantaneously. At time zero R(t) will be one, therefore F·R(t) = F. Thus, by establishing F·R(t) we can obtain an estimate for the flow. The mathematical models used here for representing of the R(t) include series of B-spline functions [3], auto-regressive moving average model (ARMA [4]), series of exponential functions and Fermi function modeling.

### **Fermi function modeling**

The use of the Fermi function was motivated by the observed similarity between the simulated impulse response for an intravascular tracer and the shape of the Fermi function. The mathematical model used for representation of the R(t) is Fermi function. Wilke et al. [5] fitted time curves for tissue impulse response function to the Fermi function with the following analytical expression

$R\left( t \right)= \frac{1}{1+e^{-\frac{t-\omega}{\tau}}} (3)$

where ω and τ are the free parameters of the model and do not have a direct physiological interpretation. Using a least squares fitting approach (e.g., a Marquardt-Levenberg nonlinear least square algorithm [5]), the parameters of equation 3 can be optimized to fit the observed data and establish a best estimate for the impulse response function and thus the flow.

### **Saturation correction**

For CA doses of 0.05 mmolkg^-1^ MRI signal intensity does not vary linearly with concentration. Signal intensity begins to saturate at higher CA concentrations causing a blunting of the IF peak and a subsequent over estimate of MBF. We used the method described by Larsson *et al*. [6] and validated by Fritz-Hansen [7] to convert signal intensity to concentration, therewith correcting for this signal saturation. The fundamental assumption is that change in longitudinal relaxation rate T1 due to a given concentration C(t) of contrast agent at time t can be written as follows

$$C(t)\cdot r_{1}= \left( \frac{T1\left( 0 \right)-T1(t)}{T1\left( t \right)\cdot T1(0)} \right) (4)$$

where T1(0) is the relaxation time without the CA, T1(t) is the relaxation time with the CA and r_1_ is the CA relaxivity. Thus, the concentration C(t) of CA at time t can be expressed as

$$C\left( t \right)= \frac{\Delta R_{1}}{r_{1}} (5)$$

Signal intensity S and T1 are related by the MR signal equation as follows

$$S= \Psi\cdot f(T1)$$

If the native, T1 of the blood before application of contrast material is known, then the value of Ψ can be calculated from the pre-contrast, baseline blood signal intensity. Similarly, the myocardial T1 values can be calculated from the myocardial signal intensity, if it is assumed Ψ that does not change between the blood and the myocardium. f(T1) can be expressed as follows

$$f\left( T1 \right)=1- e^{-R1\cdot TI}\left[ E\cdot\cos\left( \alpha\right) \right]^{n-1}+\left( 1-E \right)\frac{1-a^{n-1}}{1-a} (6)$$

where E=e-^R1·TR^, TI denotes here the delay time between the non-slice selective 90° pulse and the start of the acquisition. An analytical solution to equation 6 for T1 is not possible so it is solved using numerical methods. In our implementation a single variable non-linear zero finding algorithm was used, implemented in Octave [8]. With α=15°, TR=2.28 ms, TI =10 ms, can be equation 6 numerical approximated by

$f\left( T1 \right)^{'}= \frac{\mathrm{TR}}{\mathrm{TI}}\frac{1}{1-cos(\alpha)}-\frac{\mathrm{TR}}{R1} cos(\alpha)$ (7)

It was previously shown that, starting from this approximation for the noisy gradient echo signal, the effects of water turbulence due to water motion are minimized when (TRcos(α)) /( T1(1−cos(α))) << 1. Equation 6 can be approximated by

$$S^{'}= \Psi\cdot\left( \frac{\mathrm{TR}}{T1}\cdot\frac{1}{1-cos(\alpha)- \frac{\mathrm{TR}}{T1}cos(\alpha)} \right) (8)$$

Larsson et al. [7] used a pre-contrast T1, measurement to calculate Ψ from equation 6 is then assumed constant throughout the dynamic acquisition. Thus, T1 values for images post-contrast agent arrival can be calculated from equation 8 which can then be used to calculate C(t) concentration from equation 5. Than T1 can be evaluated with the following analytical expression:

$$T1^{'}=TR\left( \frac{\Psi}{S'(1-cos(\alpha))}+\frac{cos(\alpha)}{1-cos(\alpha)} \right) (9)$$

T1’ values these can then be used to calculate concentration from equation 5.

### Deconvolution Analysis

The Fourier convolution theorem states that convolution in the time domain is equivalent to pointwise multiplication of the Fourier transforms of the two quantities in the convolution integral equation 2.

$$\mathfrak{F}\left[ C_{m}\left( t \right) \right]\mathfrak{= F}\left[ MBR\cdot R\left( t \right)\cdot C_{b}(t) \right]\mathfrak{= F}\left[ MBR\cdot R\left( t \right) \right]\mathfrak{\cdot F[}C_{b}(t)]$$

Than

$\mathrm{MBF}=\mathfrak{F}^{-1}\left[ {\mathfrak{F}[C_{m}]}/{\mathfrak{F}[C_{b}]} \right] (11)$

This would mean that the inverse Fourier transform of the division C_m_/C_b_ is a mathematically unstable approach for calculating the impulse response from the measured blood and myocardial contrast enhancement. However, the ratio C_m_/C_b_ measures the level of signal intensity, which is assumed to be linearly proportional to concentration of a contrast agent, C_m_, in a myocardial region of interest (ROI). This concentration depends on perfusion rate and arterial concentration of the agent, C_b_, which is also acquired in the form of signal intensity in the process, through the convolution integral equation 2, where R(t) is the response function of the myocardial ROI and characterizes its perfusion properties (i.e. MBF). The goal of perfusion MRI post processing is to recover R(t) from observed C_m_, and C_b_. The most common technique is to find R(t) through solving a least square minimization problem [10]

$\min_{R}\left( t \right)\left\| C_{m}-IRF \right\|^{2}$ (12)

Although estimating MBF values from the available mathematical models have been reported in several studies, rarely they have investigated the influence of model parameters changes on the outcome of deconvolution. The mathematical model used here for representation of the R(t) is Fermi function figure 1.


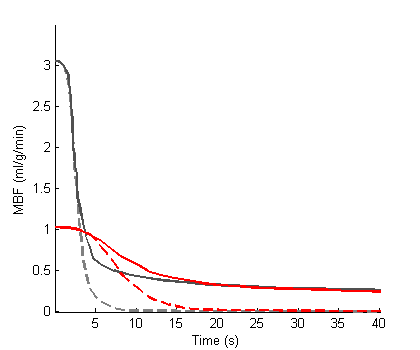


Figure 1: Examples of impulse responses of the Fermi function.

Only the amplitude of IRF(t) for t = 0 has a physiological meaning: it corresponds to the blood flow, according to the Central Volume Theorem

$$IRF\left( t=0 \right)= \frac{MBF}{1+e^{-\omega/\tau}} (13)$$

where ω defines the width of the initial plateau, before the function decays at a rate set by the parameter τ and MBF corresponds to the blood flow.

References

[1] M. Jerosch-Herold, a E. Stillman, and N. Wilke, “Magnetic resonance quantification of the myocardial perfusion reserve with a Fermi function model for constrained deconvolution.,” *Med. Phys.*, vol. 25, no. 1, pp. 73–84, 1998.

[2] M. Jerosch-Herold, N. Wilke, Y. Wang, G. R. Gong, a M. Mansoor, H. Huang, S. Gurchumelidze, and a E. Stillman, “Direct comparison of an intravascular and an extracellular contrast agent for quantification of myocardial perfusion. Cardiac MRI Group.,” *Int. J. Card. Imaging*, vol. 15, no. 6, pp. 453–464, 1999.

[3] N. Zarinabad et al. Voxel-wise quantification of myocardial perfusion by cardiac magnetic resonance. Feasibility and methods comparison. Magnetic resonance in medicine : official journal of the Society of Magnetic Resonance in Medicine, Society of Magnetic Resonance in Medicine. 2012; 68(6):1994–2004.

[4] B. Neyran et al. Mapping myocardial perfusion with an intravascular MR contrast agent: robustness of deconvolution methods at various blood flows. Magnetic resonance in medicine : official journal of the Society of Magnetic Resonance in Medicine / Society of Magnetic Resonance in Medicine. 2002;48(1):166–79.

[5] N. Wilke, M. Jerosch-Herold, Y. Wang, Y. Huang, B. V Christensen, a E. Stillman, K. Ugurbil, K. McDonald, and R. F. Wilson, “Myocardial perfusion reserve: assessment with multisection, quantitative, first-pass MR imaging,” *Radiology*, vol. 204, no. 2, pp. 373–84., 1997.

[6] D.W. Marquardt, “An Algorithm for Least-Squares Estimation of Nonlinear Parameters,” *J. Soc. Ind. Appl. Math.*, vol. 11, no. 2, pp. 431–441, 1963.

[7] H. B. Larsson, T. Fritz-Hansen, E. Rostrup, L. Sondergaard, P. Ring, and O. Henriksen, “Myocardial perfusion modeling using MRI,” *Magn. Reson. Med.*, vol. 35, no. United States PT - Journal Article LG - English, pp. 716–726, 1996.

[8] T. Fritz-Hansen, E. Rostrup, P. B. Ring, and H. B. W. Larsson, “Quantification of gadolinium-DTPA concentrations for different inversion times using an IR-turbo flash pulse sequence: A study on optimizing multislice perfusion imaging,” *Magn. Reson. Imaging*, vol. 16, no. 8, pp. 893–899, 1998.

[9] R. P. Brent, “Some Efficient Algorithms for Solving Systems of Nonlinear Equations,” *SIAM J. Numer. Anal.*, vol. 10, pp. 327–344, 2007.

[10] N. Zarinabad, A. Chiribiri, G. L. T. F. Hautvast, M. Ishida, A. Schuster, Z. Cvetkovic, P. G. Batchelor, and E. Nagel, “Voxel-wise quantification of myocardial perfusion by cardiac magnetic resonance. Feasibility and methods comparison.,” *Magn. Reson. Med.*, vol. 68, no. 6, pp. 1994–2004, 2012.
